# Supplementary material for: Polycomb-mediated silencing of miR-8 is required for maintenance of intestinal stemness in Drosophila melanogaster
Source: Nat Commun. 2024 Mar 2;15:1924. doi: 10.1038/s41467-024-46119-9 (PMC10907375; doi:10.1038/s41467-024-46119-9)
Supplement: Supplementary file 1 — Supplementary Information [file 41467_2024_46119_MOESM1_ESM.pdf]

Polycomb-mediated silencing of *miR-8* is required for maintenance of intestinal stemness in *Drosophila melanogaster*

by

Zoe Veneti, Virginia Fasoulaki, Nikolaos Kalavros, Ioannis S. Vlachos, Christos Delidakis and Aristides G. Eliopoulos

Supplementary Figures

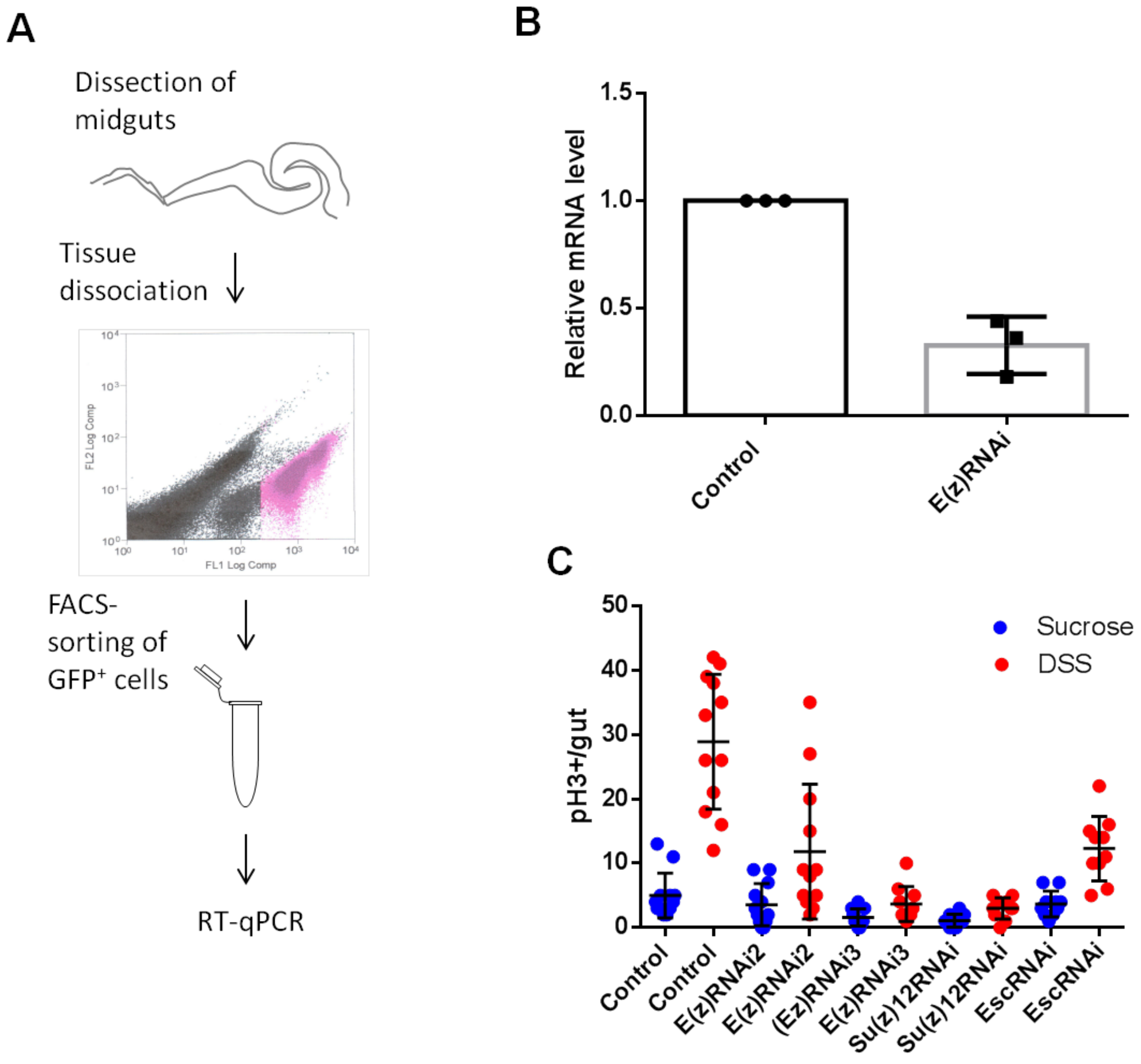

Supplementary Figure 1. FACS sorting of Drosophila esg^{ts}>GFP intestinal cell population (protocol adapted from ref.[63]). **(A)** After dissection of midguts, tissue was disrupted by enzymatic treatment with elastase and the cell suspension was FACS sorted. Cells were gated based on GFP expression and viability. RNA was isolated from the FACS-sorted cells followed by cDNA synthesis and RT-qPCR. **(B)** RT-qPCR-based confirmation of E(z) knock-down normalized to rp49. Mean and SD are shown from three independent experiments. Flies of the w^{1118} line were crossed to esg^{ts}>GFP to generate the respective control genotype. **(C)** Quantification of pH3^{+} cells of the indicated UAS-RNAi lines driven by esg-Gal4. Mean and SD are shown for 12 guts for control and E(z)-RNAi2 and 10 guts for E(z)-RNAi3, Su(z)12-RNAi and Esc-RNAi lines. Flies of the w^{1118} line were crossed to esg^{ts}>GFP to generate the respective control genotypes.

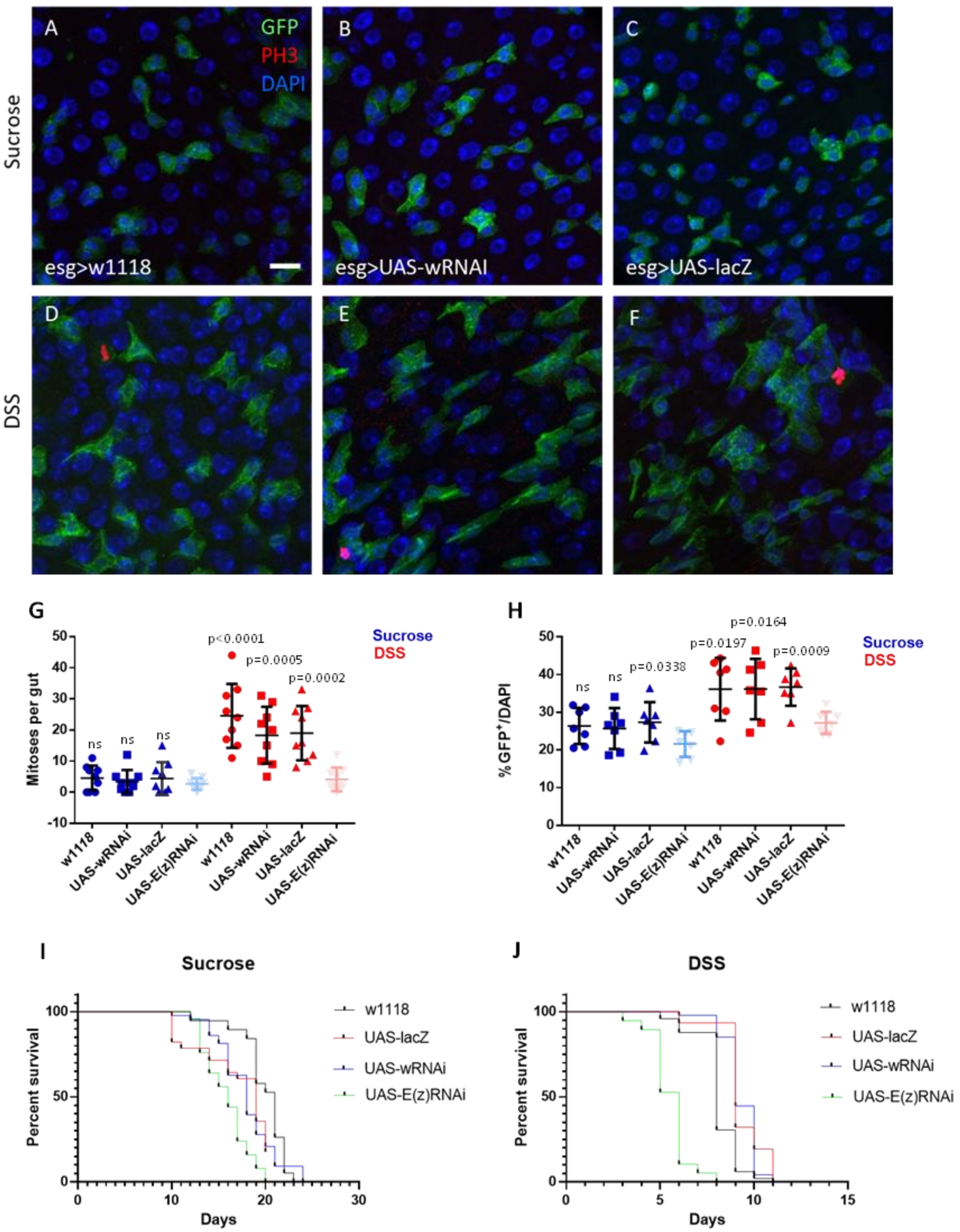

Supplementary Figure 2. Validation of the specificity of the E(z)-RNAi mediated phenotypic changes by additional controls. To exclude the possibility that "dilution" of Gal4 molecules impacts the observed phenotypes when more than one UAS targets are present, we compared w^{1118} flies **(A,D)** to either UAS-wRNAi **(B,E)** or UAS-lacZ **(C,F)** under the same driver combination (esg-Gal4, tubGal80ts, UAS-GFP). Flies of the three genotypes were treated as described in the legend of Figure 1. Both UAS-wRNAi and UAS-lacZ lines showed no statistically significant differences to w^{1118} with respect to $pH3^+$ mitotic cells **(G),** the percentage

of GFP$^+$ progenitors **(H)**, and survival upon sucrose **(I)** or DSS-fed conditions **(J)**. For (G) and (H), each control line was compared to E(z)-RNAi using two-tailed unpaired t-test (n=9 for pH3$^+$ cells and n=7 for GFP$^+$ cells per genotype). Mean and SD are shown for each line. Survival curves for control lines and E(z)-RNAi flies fed with sucrose or DSS ($p<0.0001$) were compared by the log-rank [Mantel-Cox] test (n=19 for w1118, n=28 for UAS-lacZ, n=43 for UAS-wRNAi and n=25 for E(z)-RNAi for flies fed with sucrose, n=49 for w1118, n=31 for UAS-lacZ, n=47 for UAS-wRNAi and n=19 for E(z)-RNAi control flies fed with DSS). Scale bar; 20µm, applied to images A-F.

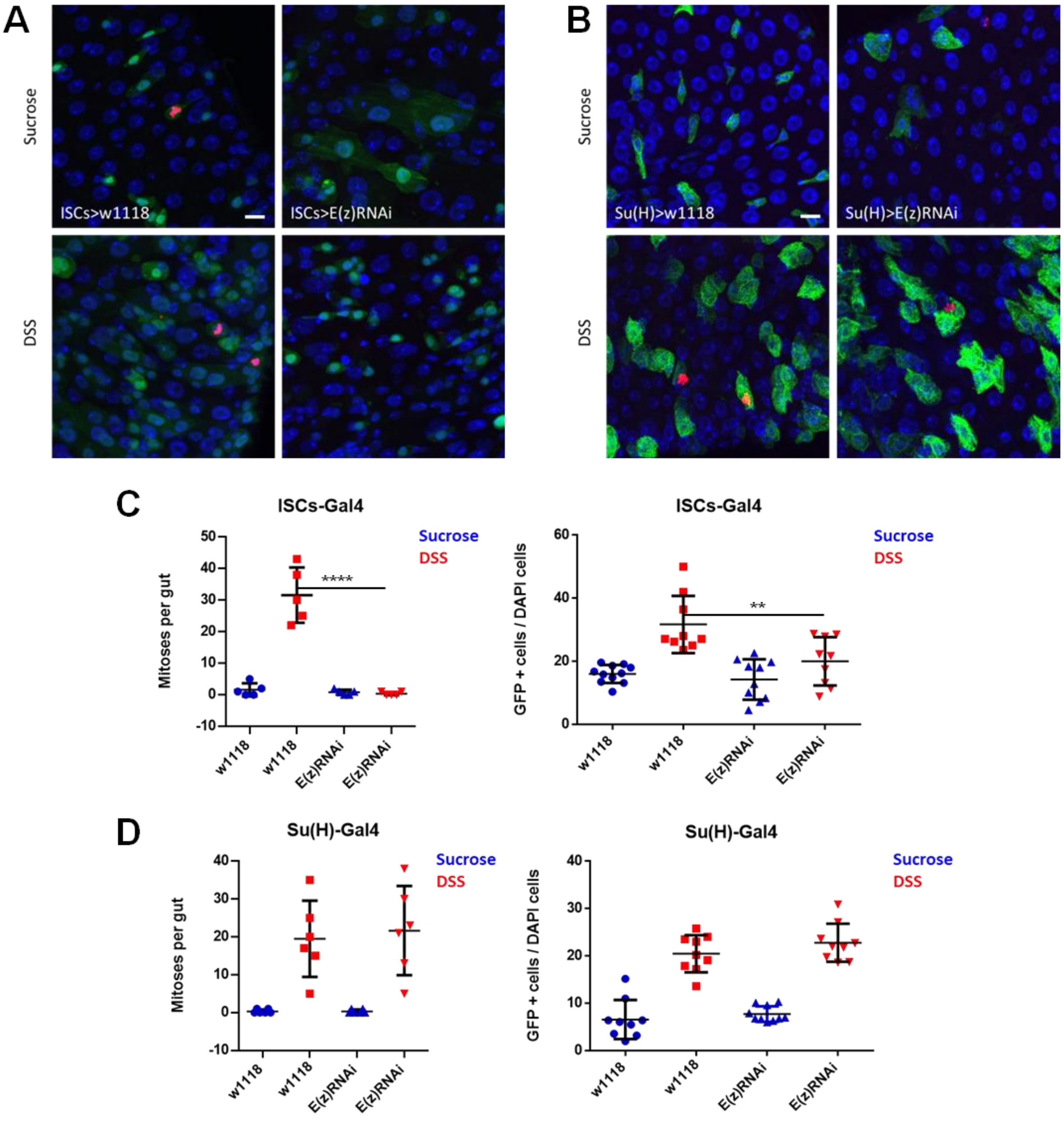

Supplementary Figure 3. E(z) functions predominantly in ISCs to regulate intestinal progenitor proliferation. E(z) was knocked down by ISC-specific Gal4 (esg-Gal4, UAS-GFP; Su(H)GBE-Gal80, tub-Gal80ts/UAS-E(z)RNAi; [26]; **A** & **C**) or EB-specific Gal4 (Su(H)GBE-Gal4, UAS-GFP; tub-Gal80ts/UAS-E(z)RNAi; [25]; **B** & **D**). Flies were fed as in Figure 1. **(A & B)** Representative immunofluorescence pH3 staining (red) of posterior midguts from young (7 day old) ISC-E(z)-RNAi or Su(H)-E(z)-RNAi female flies and their respective w^{1118} controls, fed with 5% sucrose solution or 3% DSS for 2 days, as indicated; Scale bar; 20µm for all images. **(C & D)** Quantification of $pH3^+$ positive cells representing mitotic ISCs and of GFP^+ cells as a percentage of total DAPI-stained cells in control and E(z)-RNAi midguts of the indicated genotypes and feeding conditions. (**p=0.0093; ****p<0.0001, two-tailed unpaired t-test). Data are represented as mean values ±SD.

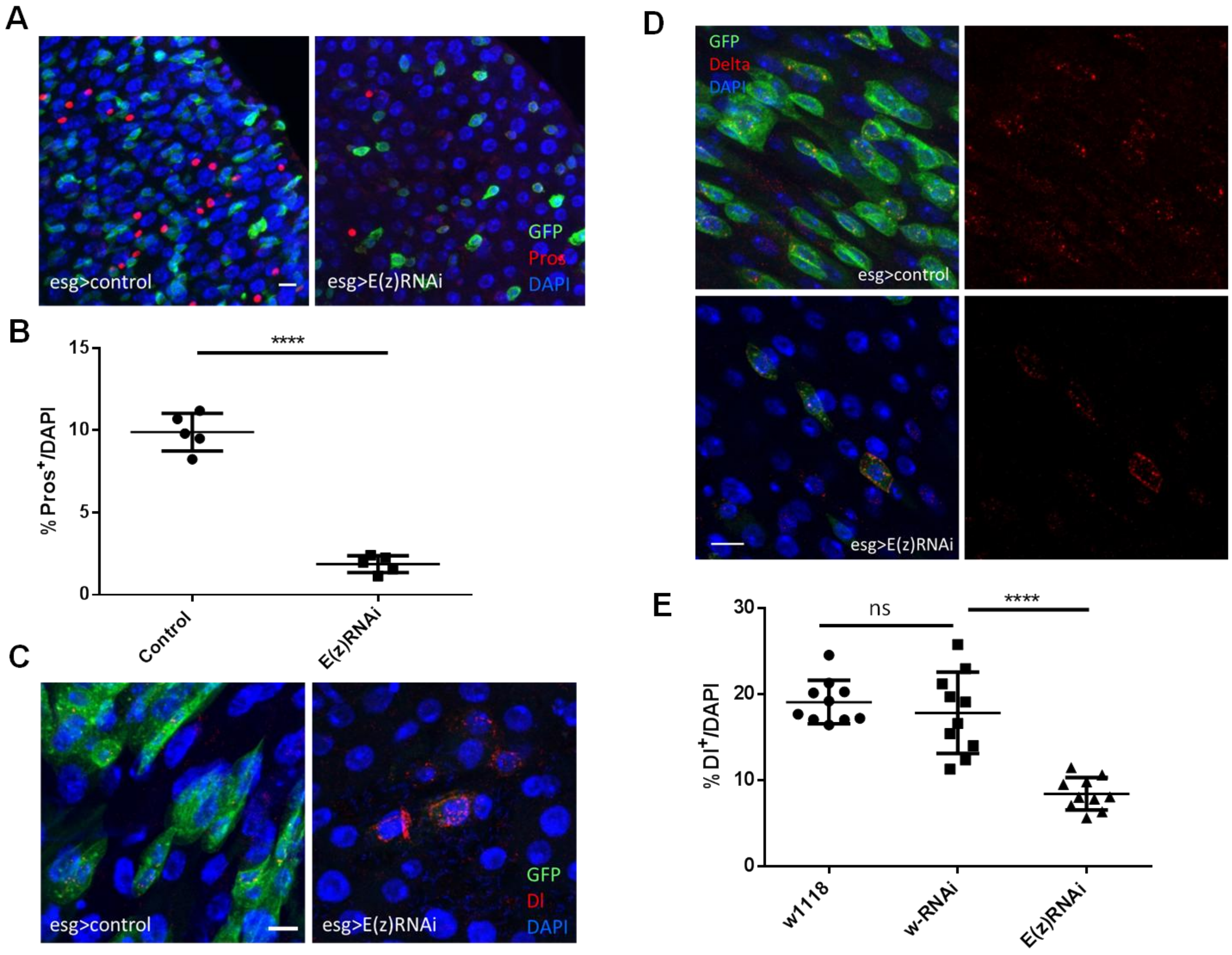

Supplementary Figure 4. (A) Staining of control and E(z)-RNAi posterior midguts expressing esg-Gal4 driven GFP (green) for the EE marker Prospero (Pros, nuclear red) and DAPI (blue) at 15 days of RNAi induction. Scale bar; 20 μm. **(B)** Quantification of $Pros^{+}$ cells as percentages of total DAPI-stained cells, at 15 days of RNAi induction. Two-tailed unpaired t-test: $p<0.0001$. Two representative, randomly selected images from posterior midguts were analyzed of n=5 flies per genotype. Data are represented as mean values ±SD. Flies of the w^{1118} line were crossed to esg^{ts}>GFP to generate the respective control genotype. **(C)** Pathology of aged midguts expressing GFP and E(z)-RNAi under the control of the esg-Gal4 driver stained with Dl antibody (red). Note the increased number of GFP^{+} progenitor cells in control flies and the presence of a few Dl^{+} intestinal stem remnants of large nuclear size and traces of esg-driven GFP. Also note the presence of bacterial DNA as revealed by small dots of DAPI staining in the E(z)-RNAi panel. Scale bar; 10μm. **(D)** Delta immunostaining of esg^{ts}>GFP flies crossed to either of two control lines (w^{1118} and w-RNAi) or E(z)-RNAi, following 10 days of RNAi induction (Scale bar; 20 μm). **(E)** Quantification of Dl^{+} cells as percentages over total DAPI-stained cells (n=10 posterior midguts per genotype; two-tailed unpaired t-test: $p<0.0001$). Note that no statistically significant difference was observed between the two control lines. Data are represented as mean values ±SD.

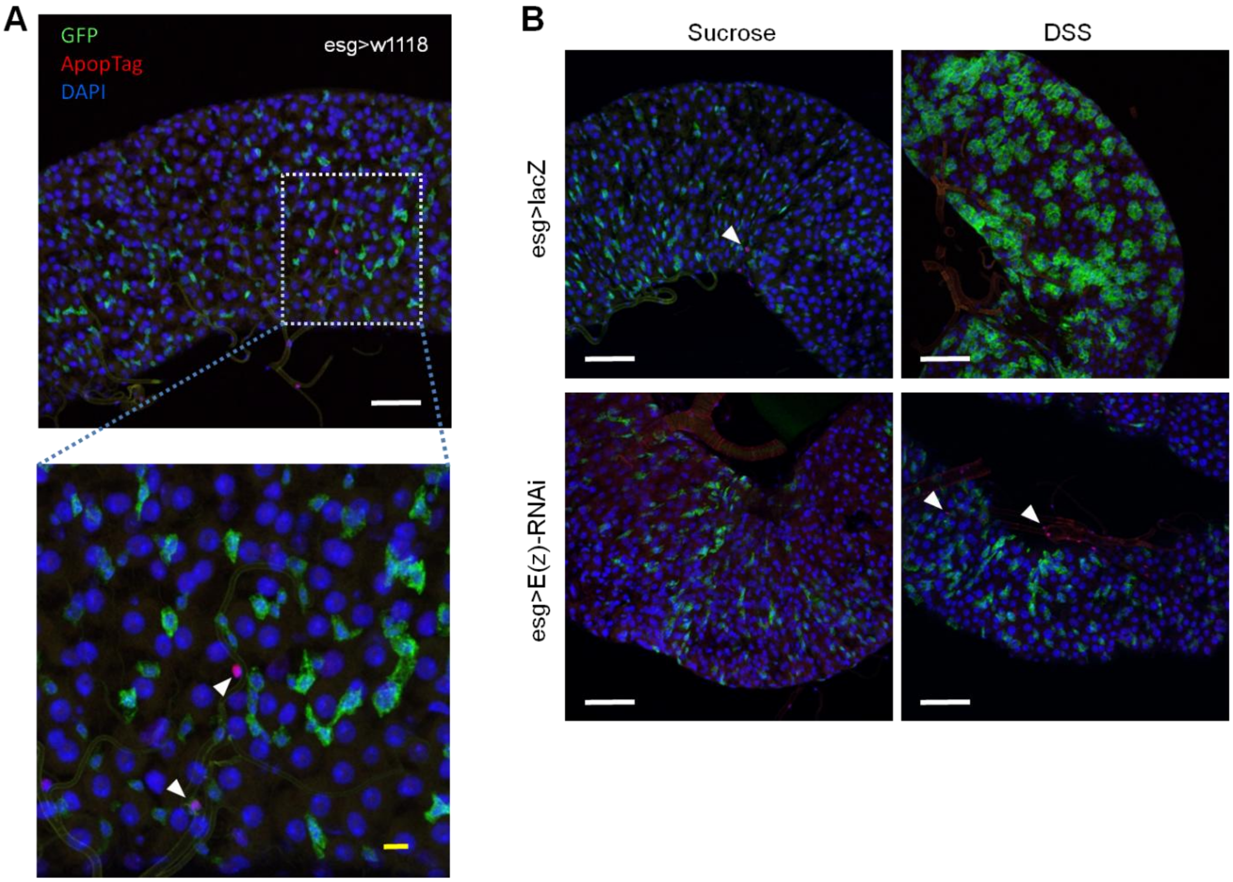

Supplementary Figure 5. The reduction in ISC numbers ensued by E(z) depletion does not involve cell death induction. Images show posterior midguts of 7 days old female w^{1118} (A), UAS-LacZ or E(z)-RNAi flies (B) expressing esg-Gal4 driven GFP (green), stained with DAPI (blue) and Apoptag (red) to detect dead cells. Images are representative of n=15 flies per genotype and treatment (sucrose vs DSS) analyzed in three independent experiments where a randomly selected posterior midgut (R4) region was examined per fly. Quantification of $ApopTag^+$ vs GFP^+ cells showed absence of apoptosis in w^{1118}, LacZ-RNAi or E(z)-RNAi progenitors irrespective of feeding conditions. Occasional apoptotic cells were noted among the GFP^+ population, and served as internal controls for the ApopTag reaction (arrowheads). White scale bars: 40µm; yellow scale bar: 20µm.

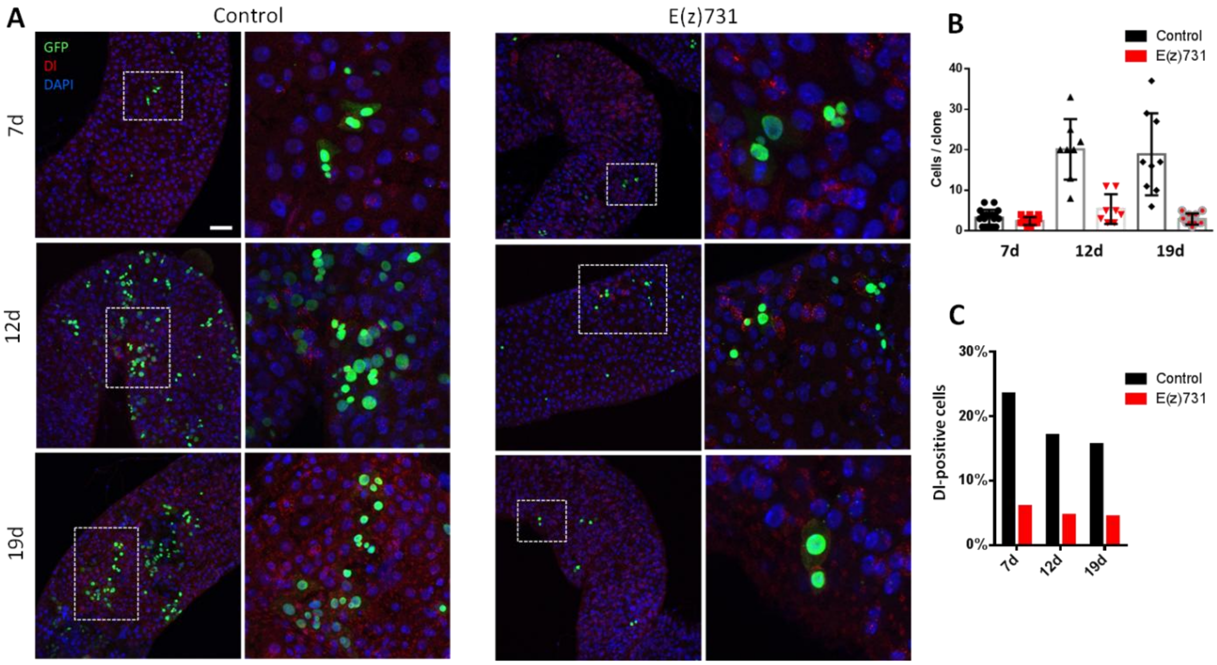

Supplementary Figure 6. (A) Time-course analysis of control and E(z)^{731} mutant MARCM clones immunostained with Delta (Dl) antibody at 7, 12 and 19 days after clonal induction. **(B)** Quantification of cell numbers per clone of the indicated genotypes and time-points. The number of the GFP labeled cells in each clone was determined for 3-5 guts and 8–20 clones for each timepoint. **(C)** Dl^{+} cell counts are plotted as percentages over total GFP positive cells. E(z)^{731} mutant MARCM clones retain their small size and contain a severely reduced amount of Dl+ cells compared to control during all time-points. Scale bar; 30μm.

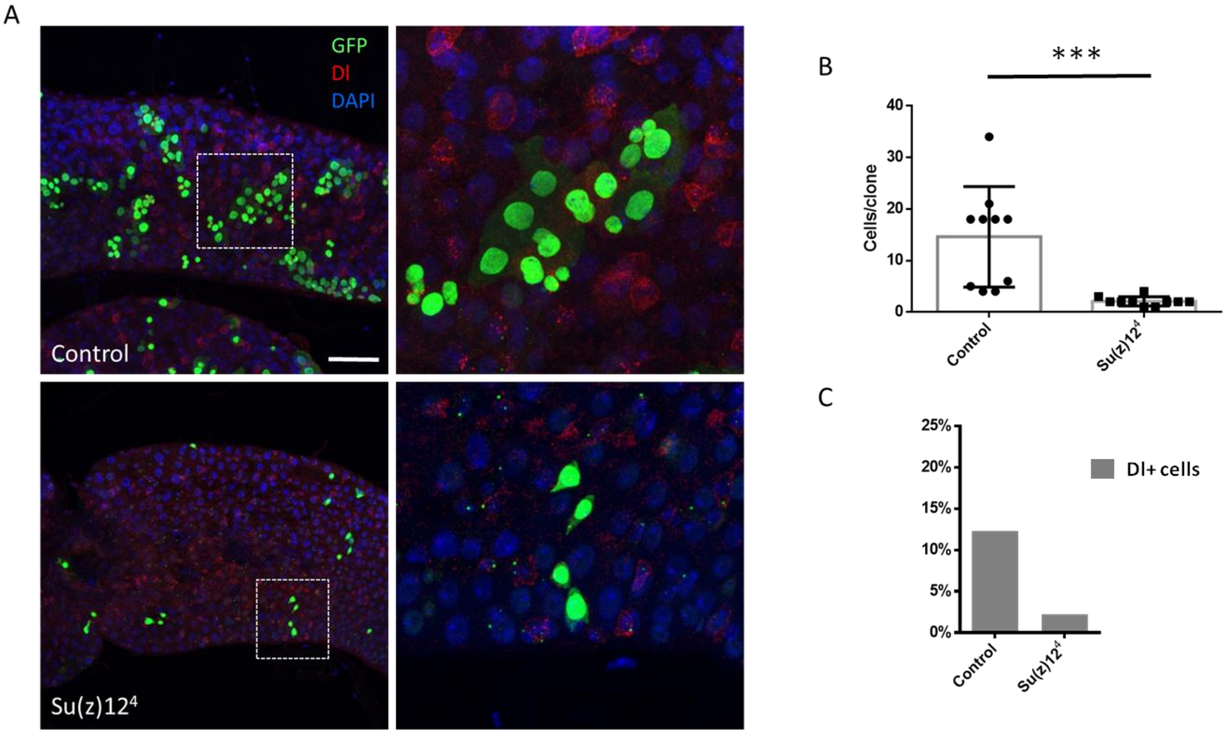

Supplementary Figure 7. (A) Immunofluorescence staining of control and Su(z)12^4 mutant MARCM clones with anti-Delta (Dl) Ab. **(B)** Quantification of cell numbers of the control and null Su(z)12^4 allele-carrying clones. Results shown are the means ± SD of n=10 for control and Su(z)12^4 clones (***p=0.0008, two-tailed unpaired t-test). **(C)** Quantification of Dl^+ vs GFP^+ cells in the midguts of control and null Su(z)12^4 allele-carrying flies. Data are shown as percentages of Dl^+ vs GFP^+ cells (n=146 GFP^+ cells/10 MARCM clones for control and n=44 GFP^+ cells/20 clones for Su(z)12^4). Scale bar; 50μm.

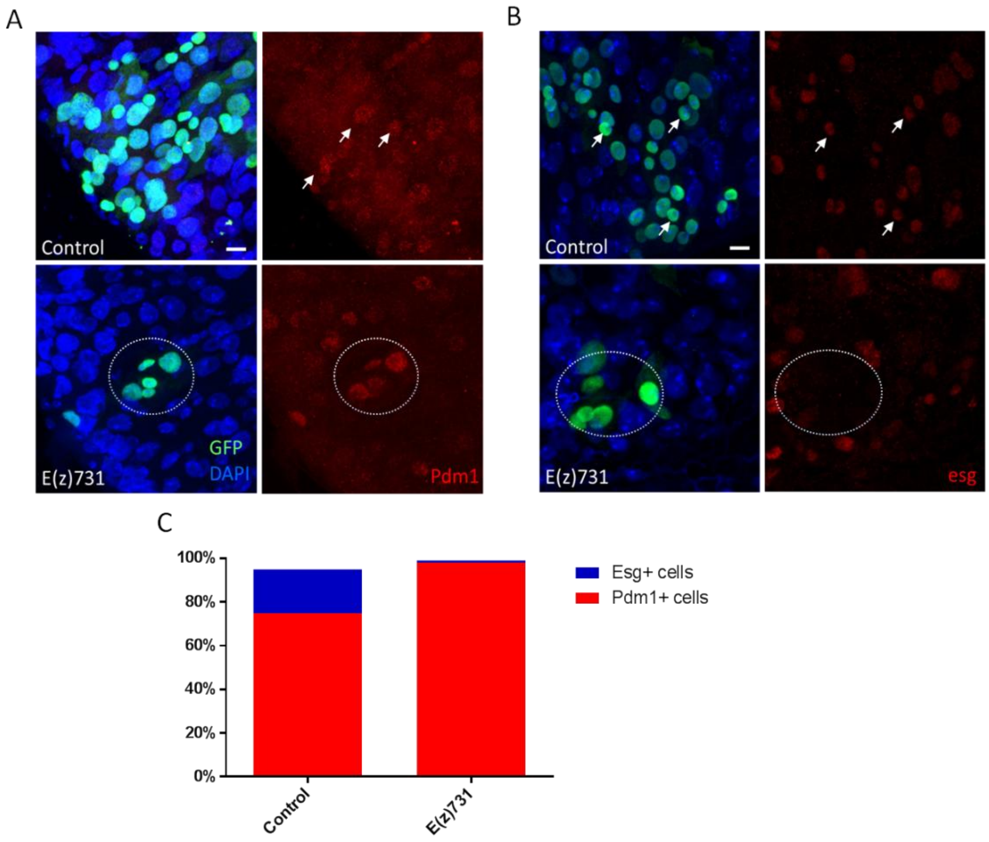

Supplementary Figure 8. Adult midguts containing nuclear localized GFP-labeled control MARCM clones or clones of the catalytically inactive allele $E(z)^{731}$ were stained for DAPI (blue) and Pdm1 (red) (A) or esg (B). Guts were dissected from the adult flies 10d after clone induction. Note that cells of mutant E(z) clones stain for Pdm1 (oval area, arrows in control clones) but not for esg (oval area, arrows in control clones), indicating acquisition of EC fate and absence of stemness. Scale bar; 10µm.

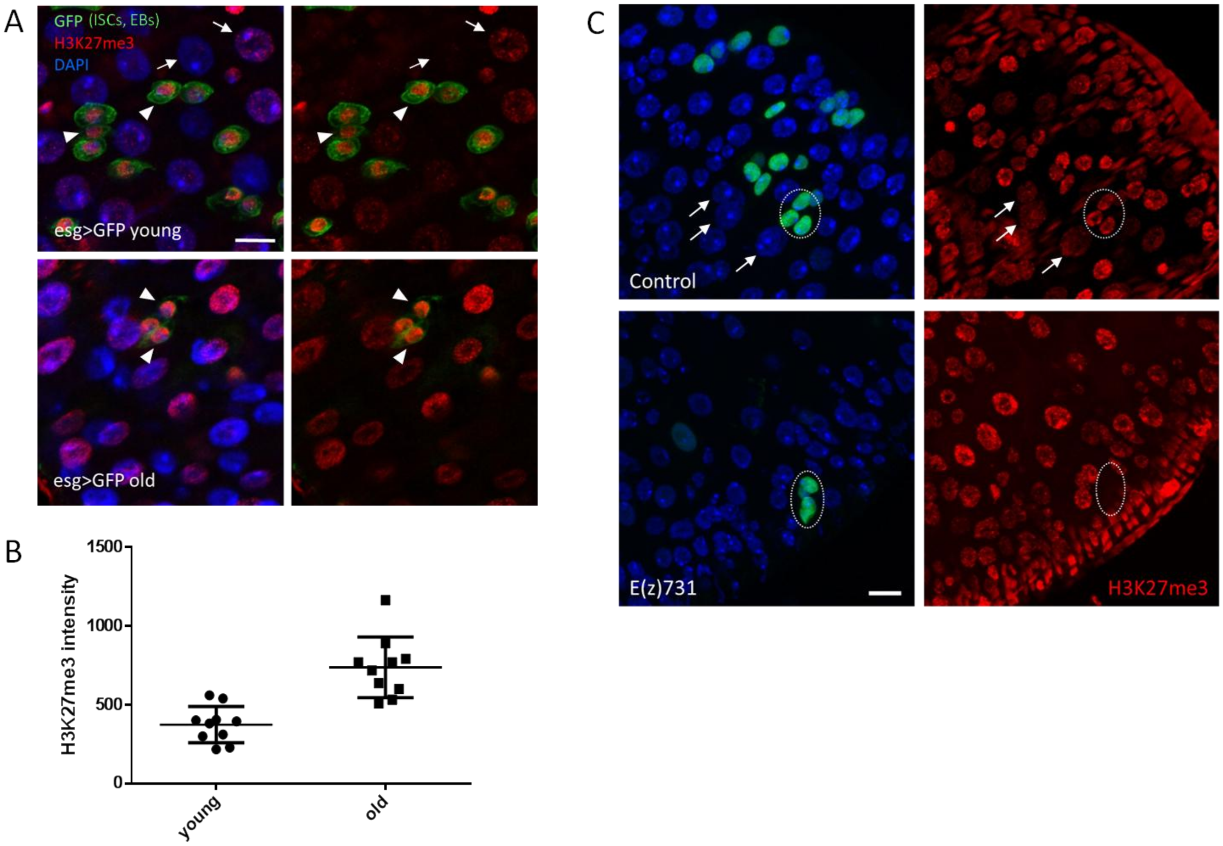

Supplementary Figure 9. Expression of H3K27me3 in Drosophila intestine is controlled by E(z). **(A)** Posterior midguts from young (2-3 days) and old (21 days) esg>GFP flies kept in 29^{0}C were immunostained for H3K27me3 and subjected to confocal microscopy. H3K27me3 is readily detected in ISCs/EBs (arrowheads) whereas expression is variable in ECs (arrows). **(B)** Quantification of the mean fluorescence intensity of young vs old ISCs. **(C)** Adult midguts containing nuclear localized GFP-labeled control MARCM clones (A) or a 3 cell clone of the catalytically inactive allele E(z)^{731} (B), stained for DAPI (blue) and H3K27me3 (red). Guts were dissected from the adult flies 7d after clone induction, stained and processed for confocal microscopy; projections from representative clones are shown. Note that cells of mutant E(z) clones (lower panel, dotted circle) are completely devoid of the methylation mark. Scale bar; 20μm.

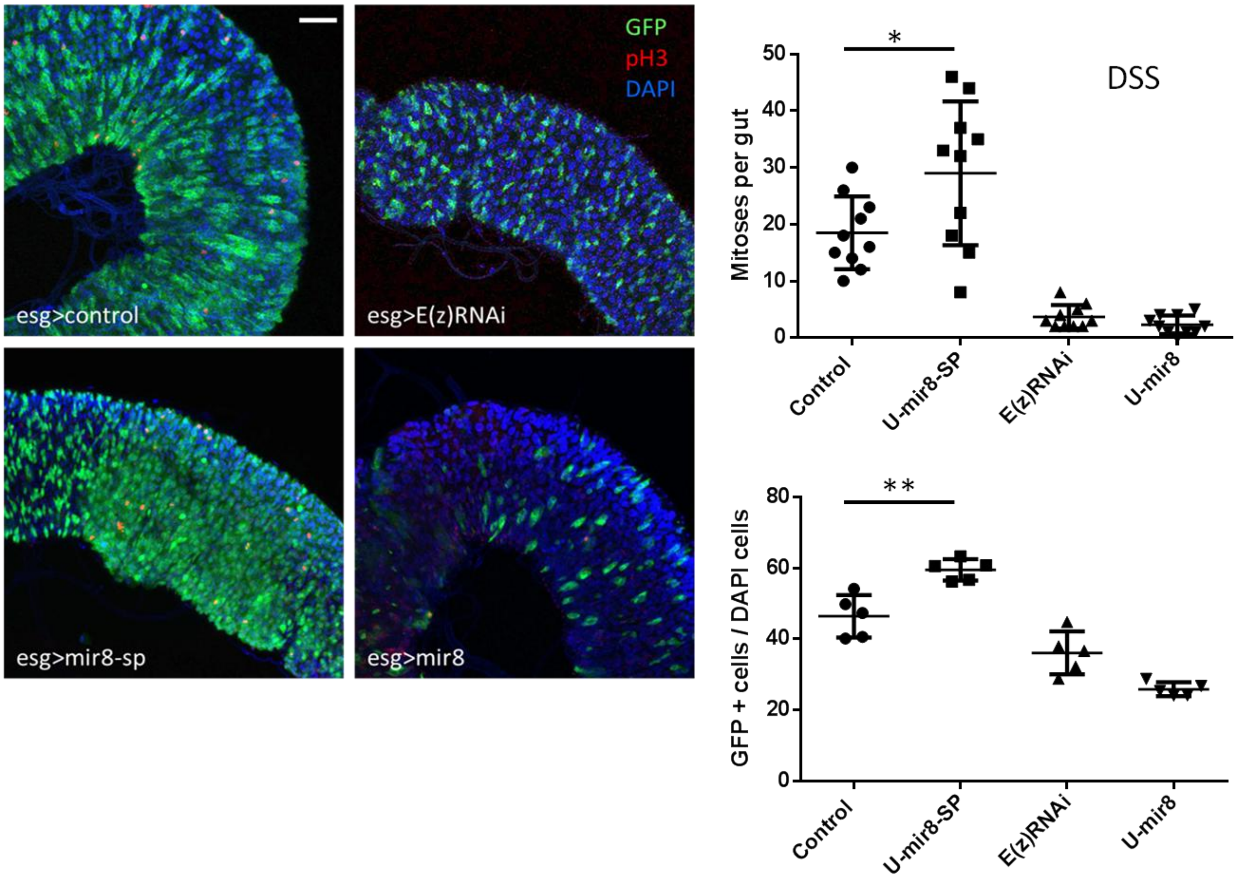

Supplementary Figure 10. Midguts of DSS fed flies expressing GFP (green) driven by esg-Gal4 were stained with DAPI (blue) and pH3 (red) to detect mitotic cells of the respective genotypes. Note that flies overexpressing *miR-8* display reduced number of $GFP^{+}/pH3^{+}$ cells, similar to that of E(z)-RNAi, while flies with reduced *miR-8* (*miR-8* sponge; sp) exhibit ISC hyperproliferation and midgut hyperplasia (*p=0.03, **p=0.0071; two-tailed unpaired t-test). Data are represented as mean values ±SD. Flies of the w^{1118} line were crossed to esg^{ts}>GFP to generate the respective control genotype. Scale bar; 50μm.

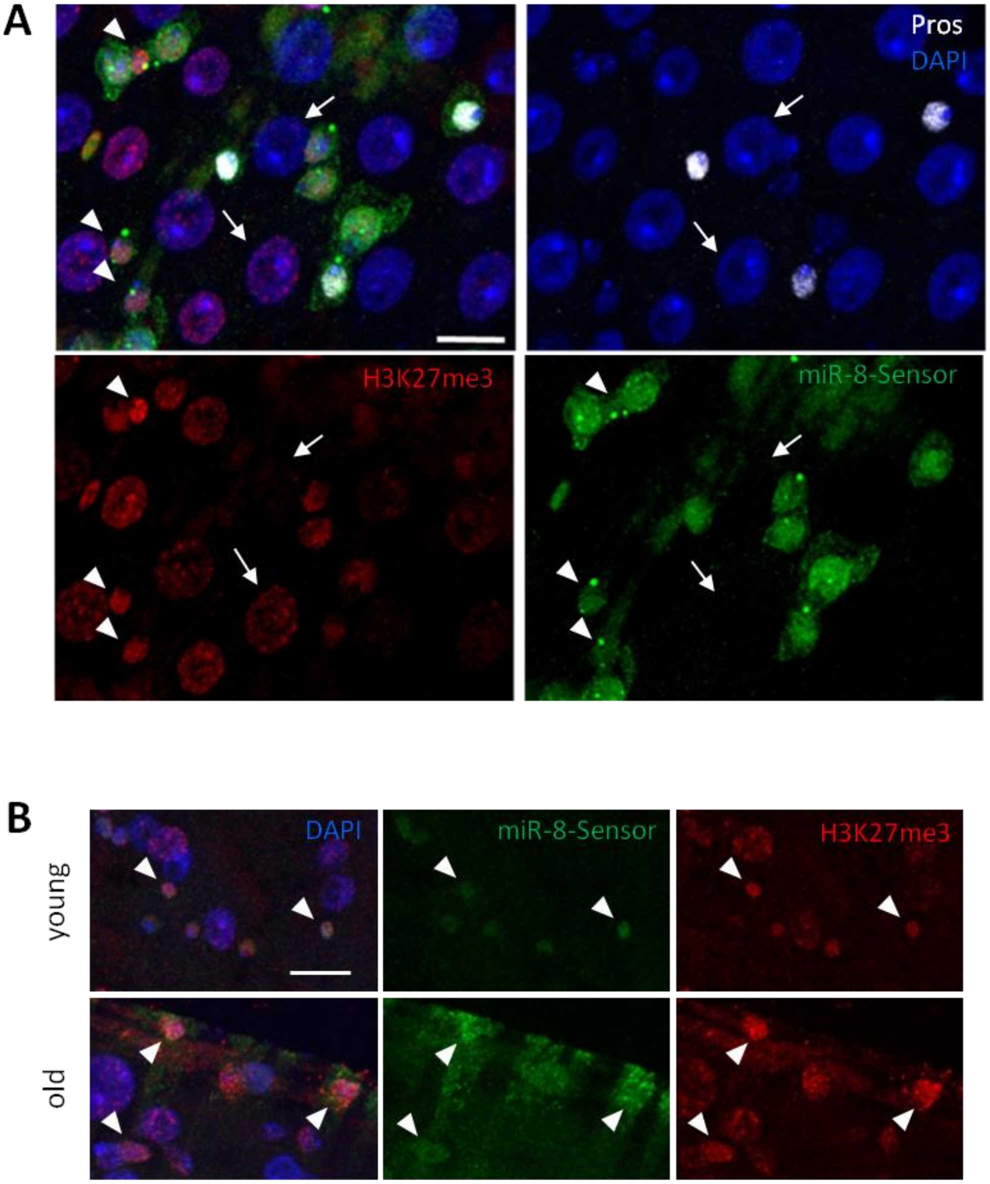

Supplementary Figure 11: Expression of H3K27me3 and *miR-8* in Drosophila midgut.
(A) Midguts of *miR-8* EGFP sensor flies were immunostained for H3K27me3 and Pros antibodies. Representative ISCs are shown by arrowheads; these cells display high levels of H3K27me3 and express EGFP reflecting low *miR-8* activity. ECs (arrows) have variable levels of H3K27me3 but never express EGFP, indicative of high *miR-8* expression.
(B) Midguts of *miR-8* EGFP sensor young (2-3 days) *versus* old (21 days) flies were immunostained for H3K27me3; arrowheads depict representative progenitor cells. Note the higher expression of *miR-8* and H3K27me3 in old versus young progenitor cells. Scale: 20µm.

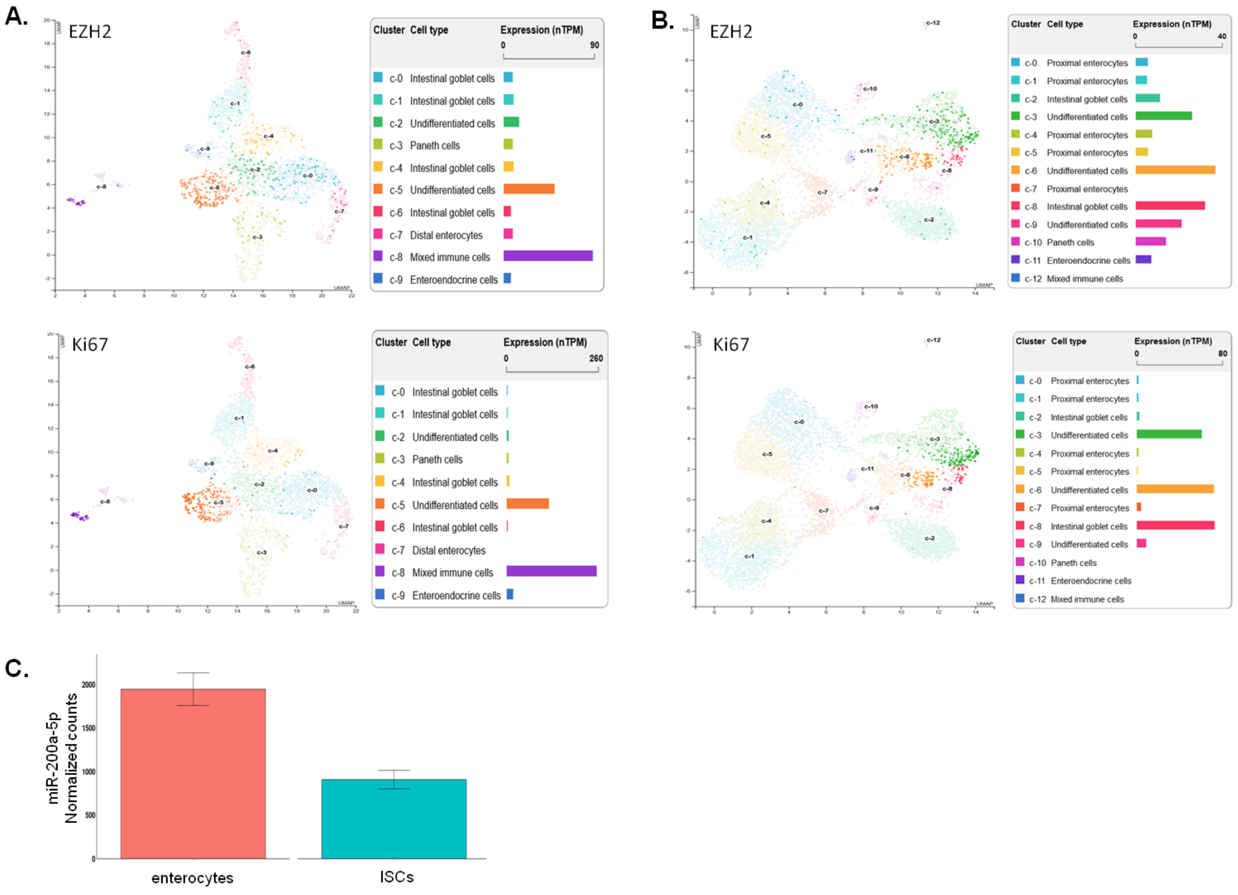

Supplementary Figure 12. *EZH2* is inversely correlated with *miR-200a* in normal human intestinal progenitors. EZH2 is predominantly expressed in highly proliferating (high Ki67 levels) undifferentiated cells in the human rectum **(A)** and small intestine **(B)**. EZH2 and Ki67 expression data was extracted from the Human Protein Atlas single cell RNAseq database. **(C)** Levels of *miR-200a-5p* are reduced in ISCs of the human small intestine compared to normal enterocytes (data extracted from ref. [32]).

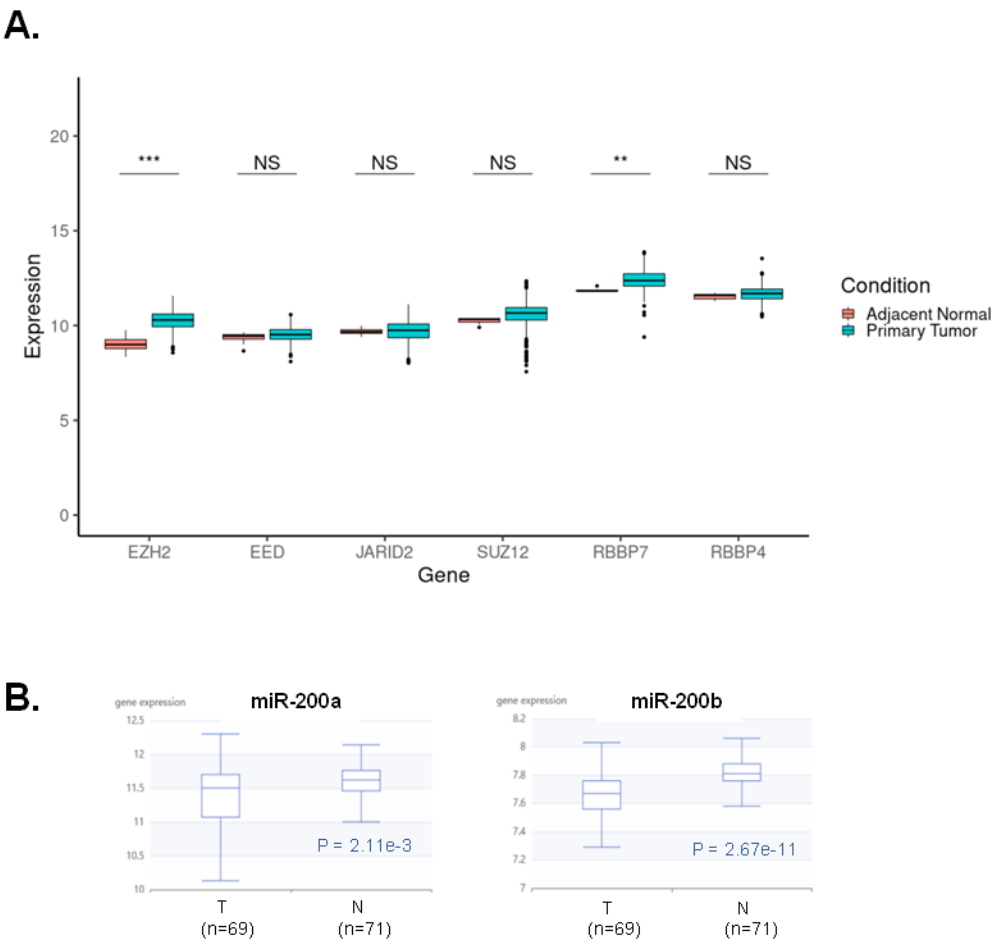

Supplementary Figure 13. Expression of PRC2.1 and PRC2.2 components and of *miR-200* in malignant vs normal human rectum.

(A) Data extracted from the Cancer Genome Atlas (TCGA) database show that compared to normal tissue, paired colorectal tumors express higher levels of EZH2 and RBBP7.

(B) Data extracted from Gaedcke *et al.* show that the levels of *miR-200a* and *miR-200b* are reduced in colorectal tumors compared to normal tissue[35].

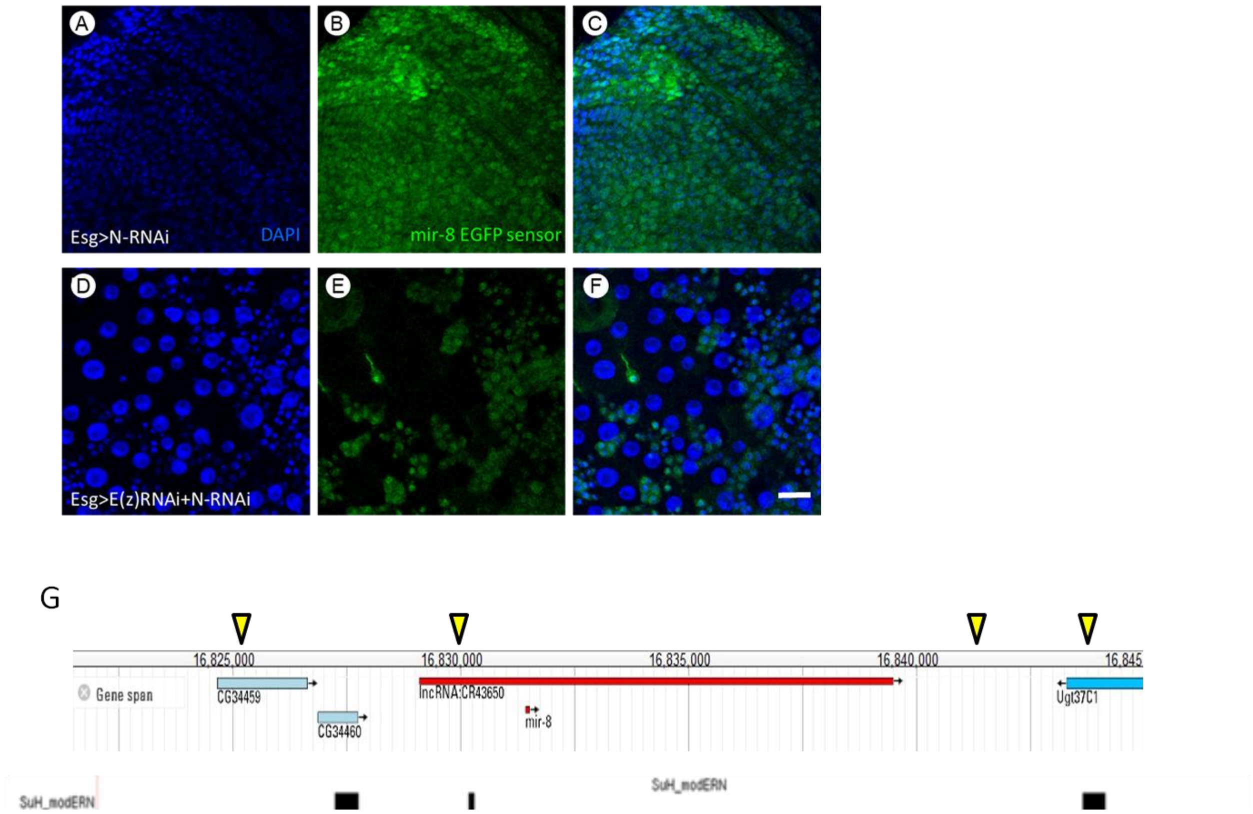

Supplementary Figure 14: Notch and E(z) impact *miR-8* expression.

(A) Flies with Notch RNAi (N-RNAi) driven by *esg*-Gal4 for 5 days display dysplasias typified by small round cells that occupy the entire posterior midgut and are all positive for the *miR-8*-EGFP-sensor, indicating decreased *miR-8* activity. Knockdown of both E(z) and Notch ameliorated the N-RNAi phenotype as smaller clusters of small round green cells were observed interspersed with large *miR-8*-EGFP sensor-negative enterocytes. Scale bar; 25µm.

(B) A genetic map of *miR-8* locus is shown with four potential Su(H) binding sites (yellow triangles) identified by FIMO (https://meme-suite.org/meme/tools/fimo) using PWM files for Su(H) from Fly Factor Survey (http://pgfe.umassmed.edu/TFDBS/). Two of the predicted binding sites were also detected by ChIP-seq of 0-24h embryo chromatin in the modERN/ MODENCODE project (https://www.encodeproject.org/experiments/ENCSR325UBZ/)[64] (lower panel).
