# Supplementary material for: Polycomb-mediated silencing of miR-8 is required for maintenance of intestinal stemness in Drosophila melanogaster
Source: Nat Commun. 2024 Mar 2;15:1924. doi: 10.1038/s41467-024-46119-9 (PMC10907375; doi:10.1038/s41467-024-46119-9)
Supplement: Supplementary file 3 — Description of Additional Supplementary Files [file 41467_2024_46119_MOESM3_ESM.pdf]

Description of Additional Supplementary Files

File Name: Supplementary Data 1
Description: Differential enrichment analysis of H3K27me3.

File Name: Supplementary Data 2
Description: Genes with decreased H3K27me3 peak values in E(z)-RNAi line.
